# Comparative analysis of complete plastid genomes from wild soybean (*Glycine soja*) and nine other *Glycine* species

**DOI:** 10.1371/journal.pone.0182281

**Published:** 2017-08-01

**Authors:** Sajjad Asaf, Abdul Latif Khan, Muhammad Aaqil Khan, Qari Muhammad Imran, Sang-Mo Kang, Khdija Al-Hosni, Eun Ju Jeong, Ko Eun Lee, In-Jung Lee

**Affiliations:** 1 School of Applied Biosciences, Kyungpook National University, Daegu, Republic of Korea; 2 Chair of Oman's Medicinal Plants & Marine Natural Products, University of Nizwa, Nizwa, Oman; Chinese Academy of Medical Sciences and Peking Union Medical College, CHINA

## Abstract

The plastid genomes of different plant species exhibit significant variation, thereby providing valuable markers for exploring evolutionary relationships and population genetics. *Glycine soja* (wild soybean) is recognized as the wild ancestor of cultivated soybean (G. *max*), representing a valuable genetic resource for soybean breeding programmes. In the present study, the complete plastid genome of *G*. *soja* was sequenced using Illumina paired-end sequencing and then compared it for the first time with previously reported plastid genome sequences from nine other *Glycine* species. The *G*. *soja* plastid genome was 152,224 bp in length and possessed a typical quadripartite structure, consisting of a pair of inverted repeats (IRa/IRb; 25,574 bp) separated by small (178,963 bp) and large (83,181 bp) single-copy regions, with a 51-kb inversion in the large single-copy region. The genome encoded 134 genes, including 87 protein-coding genes, eight ribosomal RNA genes, and 39 transfer RNA genes, and possessed 204 randomly distributed microsatellites, including 15 forward, 25 tandem, and 34 palindromic repeats. Whole-plastid genome comparisons revealed an overall high degree of sequence similarity between *G*. *max* and *G*. *gracilis* and some divergence in the intergenic spacers of other species. Greater numbers of indels and SNP substitutions were observed compared with *G*. *cyrtoloba*. The sequence of the *accD* gene from *G*. *soja* was highly divergent from those of the other species except for *G*. *max* and *G*. *gracilis*. Phylogenomic analyses of the complete plastid genomes and 76 shared genes yielded an identical topology and indicated that *G*. *soja* is closely related to *G*. *max* and *G*. *gracilis*. The complete *G*. *soja* genome sequenced in the present study is a valuable resource for investigating the population and evolutionary genetics of *Glycine* species and can be used to identify related species.

## Introduction

The chloroplast (cp) is a key organelle in photosynthesis and in the biosynthesis of fatty acids, starches, amino acids, and pigments [1, 2]. In angiosperms, plastomes are typically circular and highly conserved, ranging from 115 to 165 kb in length and comprising a small single-copy region (SSC; 16–27 kb) and a large-single-copy region (LSC; 80–90 kb), separated by a pair of inverted repeats (IRs) [3, 4]. Most plastomes also contain 110–130 genes encoding up to 80 unique proteins and approximately 4 rRNAs and 30 tRNAs. Most of the protein-coding genes are associated with photosynthesis or other biochemical processes in plant cells, such as synthesis of amino acids, sugars, vitamins, lipids, pigments, and starches, storage, nitrogen metabolism, sulphate reduction, and immune responses [5, 6]. In contrast to mitochondrial and nuclear genomes, the plastomes of plants are highly conserved in regard to gene structure, organization, and content [4]. However, gene duplications, mutations, rearrangements, and losses have been observed in some angiosperm lineages [7]. Rearrangements of plastid gene order are generally observed in taxa with plastomes that exhibit at least one of the following qualities: variable IR region size, loss of one IR region, a high frequency of small dispersed repeats, complete or near-complete lack of photosynthesis, or biparental cp inheritance [8]. In addition, plastome inversions have been reported in a number of angiosperm families, including Asteraceae [9], Campanulaceae [10], Onagraceae [11], Leguminosae [12], and Geraniaceae [13, 14]. The plastomes of several members of the Papilionoideae also exhibit significant variation and rearrangement, including the loss of an IR region [15] and inversion of a 50-kb portion of the LSC [16, 17]. These features, as well as the loss of introns from the *rps12* and *clpP* genes [18, 19] and transfer of *rpl22* to the nucleus [20, 21], have been well documented, and their occurrence has been mapped onto the phylogeny of Leguminosae [19].

The genus *Glycine* comprises at least 28 species, which are separated into two subgenera, Glycine and *Soja*. The annuals include cultivated soybean, *G*. *max*, and the wild soybean, *G*. *soja*, that are native to eastern Asia, whereas most of the other species are perennials that are native to Australia. Researchers previously classified *Glycine* species into various groups (A-I) on the basis of fertility of artificially produced hybrids and the degree to which meiotic chromosomes pair [22], and Ratnaparkhe et al. (2011) [23] further reviewed the nine genome groups using isozymes and sequences of two nuclear loci (H3D and nrDNA ITS).

Plastid data from various *Glycine* species (annual and perennial) have been used in studies of phylogenetic and genetic diversity [24–28], including the investigation of neopolyploidy [29, 30]. For example, Doyle et al. (1990b) [24] identified three major clades within the perennial subgenus, showing varying degrees of agreement with nuclear phylogenies. However, additional research revealed incongruence between the plastid and nuclear phylogenies of the various genome groups [31]. The most noticeable incongruity was the placement of *G*. *falcata*, which is the sole species in the F-genome group. According to the H3D gene-based phylogeny, *G*. *falcata* is sister to all other perennial species, whereas chloroplast DNA fragment- based phylogenies strongly supported the placement of *G*. *falcata* in the A-genome clade [16, 30, 32].

The advent of high-throughput sequencing technology has facilitated rapid progress in the field of genomics, especially in cp genetics. Since the first plastome was sequenced in 1986 [33], over 800 complete plastid genome sequences have been made available through the National Center for Biotechnology Information (NCBI) organelle genome database, including 300 from crop and tree genomes [34]. To date, complete plastomes have been reported for nine *Glycine* species [35–37]. In the present study, the complete plastome of *G*. *soja* was sequenced (GenBank accession number: KY241814) with the aim of elucidating global patterns of structural variation in the *G*. *soja* plastome and comparing it for the first time with the available plastomes of nine other *Glycine* species (*G*. *max*, *G*. *gracilis*, *G*. *canescens*, *G*. *cyrtoloba*, *G*. *dolichocarpa*, *G*. *falcata*, *G*. *stenophita*, *G*. *syndetika*, and *G*. *tomentella*).

## Materials and methods

### Chloroplast genome sequencing and assembly

The *G*. *soja* (accession KLG90379), seeds were received from the National Gene Bank of the Rural Development Administration of the Republic of Korea. Plants were cultivated in greenhouse at the Kyungpook National University, Republic of Korea. Plastid DNA was extracted from young leaves using the protocol described by Hu et al. [38], and the resulting DNA was sequenced using the Illumina HiSeq-2000 platform (San Diego, CA, USA) at Macrogen (Seoul, Korea). The *G*. *soja* plastome was then assembled *de novo* using a bioinformatics pipeline (http://phyzen.com). More specifically, a 400-bp paired-end library was produced according to the Illumina PE standard protocol, which resulted in 28,110,596 bp of sequence data, with a 101-bp average read length. Raw reads with Phred scores of 20 or lower were removed from the total PE reads using the CLC-quality trim tool, and *de novo* assembly of the trimmed reads was accomplished using CLC Genomics Workbench v7.0 (CLC Bio, Aarhus, Denmark) with a minimum overlap of 200 to 600 bp. The resulting contigs were compared against the *G*. *max* plastome using BLASTN with an E-value cutoff of 1e-5, and five contigs were identified and temporarily arranged based on their mapping position in the reference genome. After initial assembly, primers were designed (S1 Table) based on the terminal sequences of adjacent contigs, and PCR amplification and subsequent DNA sequencing were employed to fill in the gaps. PCR amplification was performed in 20-μl reactions that contained 1× reaction buffer, 0.4 μl dNTPs (10 mM), 0.1 μl Taq (Solg h-Taq DNA Polymerase), 1 μl (10 pm/μl) primers, and 1 μl (10 ng/μl) DNA, under the following conditions: initial denaturation at 95°C for 5 min; 35 cycles of 95°C for 30 s, 60°C for 20 s, and 72°C for 30 s; and a final extension step of 72°C for 5 min. After incorporating the additional sequencing results, the complete plastome was used as a reference to map the remaining unmapped short reads to improve the sequence coverage of the assembled genome.

### Analysis of gene content and sequence architecture

The *G*. *soja* plastome was annotated using DOGMA [39] and checked manually, and codon positions were adjusted based on comparison with homologs in the plastome of *G*. *max*. The transfer RNA sequences of the *G*. *soja* plastome were verified using tRNAscan-SE version 1.21 [40], with the default settings, and structural features were illustrated using OGDRAW [41]. To examine deviations in synonymous codon usage by avoiding the influence of the amino acid composition, the relative synonymous codon usage (RSCU) was determined using MEGA 6 [42]. Finally, the divergence of the new *G*. *soja* plastome from both perennial and annual *Glycine* species was assessed with mVISTA [43] in Shuffle-LAGAN mode, employing the new *G*. *soja* genome as a reference.

### Characterization of repeat sequences and simple sequence repeats (SSRs)

Repeat sequences, including direct, reverse, and palindromic repeats, were identified within the plastome using REPuter [44], with the following settings: Hamming distance of 3, ≥90% sequence identity, and minimum repeat size of 30 bp. Additionally, SSRs were detected using Phobos version 3.3.12 [45], with the search parameters set to ≥10 repeat units for mononucleotide repeats, ≥8 repeat units for dinucleotide repeats, ≥4 repeat units for trinucleotide and tetranucleotide repeats, and ≥3 repeat units for pentanucleotide and hexanucleotide repeats. Tandem repeats were identified using Tandem Repeats Finder version 4.07 b [46], with default settings.

### Sequence divergence and phylogenetic analyses

The average pairwise sequence divergence of 76 shared genes and the complete plastomes of 11 *Glycine* species were analysed using data from *G*. *soja* new (KY241814), *G*. *soja* old (NC022868), *G*. *max*, *G*. *gracilis*, *G*. *canescens*, *G*. *cyrtoloba*, *G*. *dolichocarpa*, *G*. *falcata*, *G*. *stenophita*, *G*. *syndetika*, and *G*. *tomentella*. Missing and ambiguous gene annotations were confirmed through comparative sequence analysis, after assembling a multiple sequence alignment and comparing gene order. The complete genome dataset was aligned using MAFFT version 7.222 [47], with default parameters, and Kimura’s two-parameter (K2P) model was selected to calculate pairwise sequence divergence [48]. A sliding window analysis was conducted to determine the nucleotide diversity (Pi) of the cp genome using DnaSP (DNA Sequences Polymorphism version 5.10.01) software [49]. The step size was set to 200 bp, with a window length of 800 bp. Similarly, Indel polymorphisms among the complete genomes were identified using DnaSP 5.10.01 [49], and a custom Python script (https://www.biostars.org/p/119214/) was employed to identify single-nucleotide polymorphisms. To resolve the phylogenetic position of *G*. *soja* within the genus *Glycine*, ten published *Glycine* species plastomes were downloaded from the NCBI database for phylogenetic analysis. Multiple alignment of the complete plastomes were constructed based on the conserved structure and gene order of the plastid genomes [8], and four methods were employed to construct phylogenetic trees: Bayesian inference (BI), implemented using MrBayes 3.1.2 [50]; maximum parsimony (MP), implemented using PAUP 4.0 [51]; and both maximum likelihood (ML) and joining-joining (NJ), implemented using MEGA 6 [42], employing previously described settings [52, 53]. In a second phylogenetic analysis, 76 shared cp genes from eleven *Glycine* species and two outgroup species (*Phaseolus vulgaris* and *Vigna radiata*) were aligned using ClustalX with default settings, followed by manual adjustment to preserve reading frames. Finally, the same four phylogenetic inference methods were employed to infer trees from the 76 concatenated genes, using the same settings [52, 53].

## Results and discussion

### Plastid genome organization

**Fig 1:**
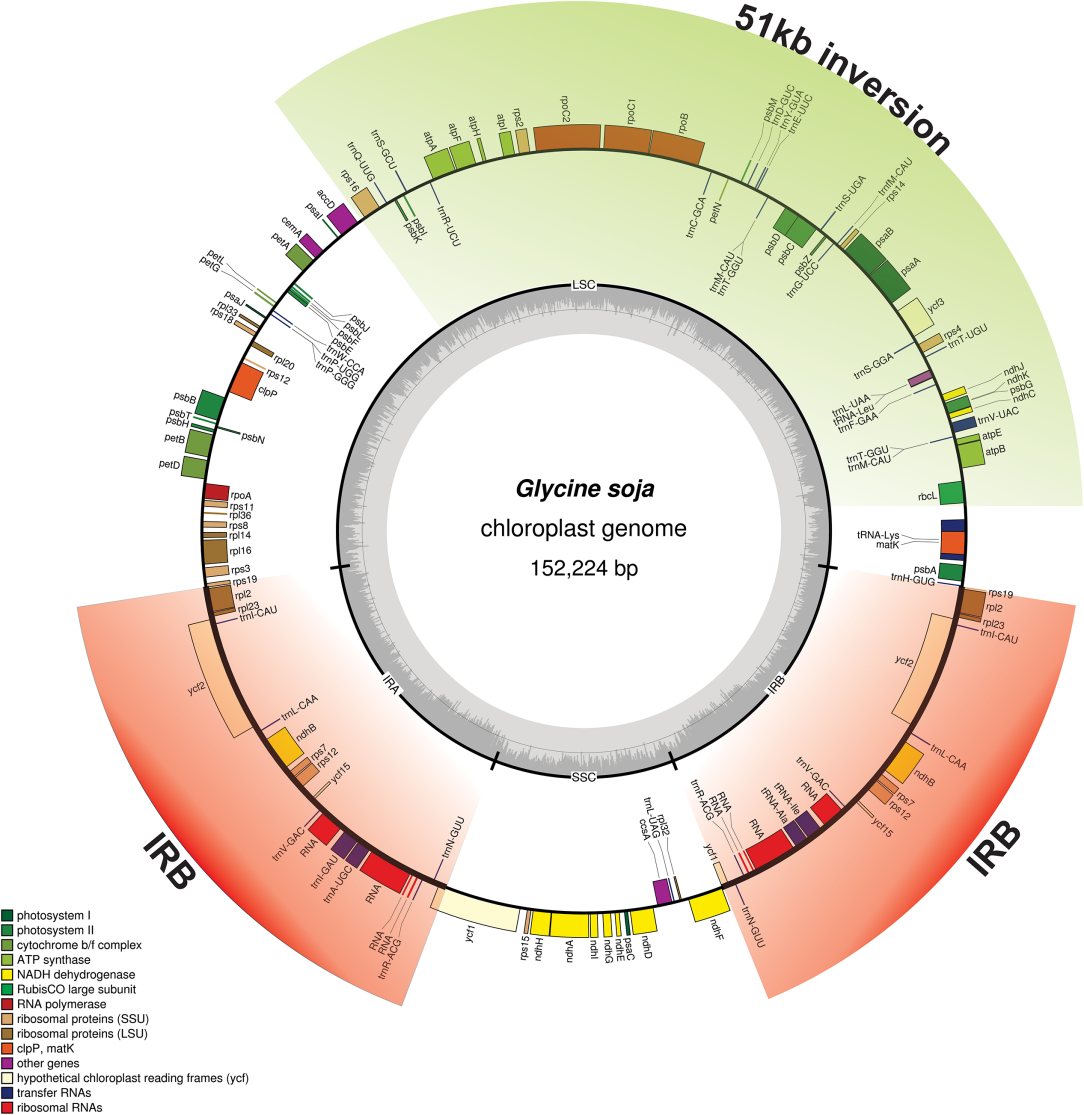
Gene map of the *Glycine soja* plastid genome. Thick lines in the red area indicate the extent of the inverted repeat regions (IRa and IRb; 25,574 bp), which separate the genome into small (SSC; 17,896 bp) and large (LSC; 83,181 bp) single-copy regions. Genes located inside the circle are transcribed clockwise, and those outside the circle are transcribed counterclockwise. Genes belonging to different functional groups are colour-coded. The dark grey in the inner circle corresponds to the GC content, and the light grey corresponds to the AT content. The green colour arc indicates the location of the 51-kb inversion.

A total of 2,611,513 reads with an average read length of 101 bp were obtained, and these reads provided 1514.9× coverage of the plastome. The consensus sequence for a specific position was generated by assembling reads that were mapped with at least 934 reads per position and was used to construct the complete sequence of the *G*. *soja* plastome. The assembled *G*. *soja* plastome of was typical of angiosperms, with a pair of IR regions (25,574 bp), an LSC of 83,181 bp, and an SSC of 178,963 bp (Fig 1); a total size of 152,224 bp; and a GC content of 35.4% (Table 1). In addition, approximately 33.23% of the genome was non-coding, whereas protein-coding, rRNA, and tRNA genes constituted 52.06, 5.94, and 1.92% of the plastome, respectively (Table 2), similar to the values observed in other legume genomes. As observed in other angiosperm plastomes, the GC content was unequally distributed in the *G*. *soja* plastome; it was high in the IR regions (41.8%), moderate in the LSC region (32.8%), and low in the SSC region (28.73%; Table 1). The high GC content of the IR regions is due to the presence of eight ribosomal RNA (rRNA) sequences in these regions, as reported previously [54, 55].

**Table 1:** Summary of complete chloroplast genomes for ten *Glycine* species.

Region	*G*.*soja* ^*a*^	*G*.*soja*	*G*.*max*	*G*.*graci*	*G*.*canes*	*G*.*cyrtol*	*G*. *doli*	*G*. *falca*	*G*. *stenop*	*G*.*syndet*	*G*. *tome*
**LSC**											
Length (bp)	83,181	83,174	83,174	83,175	83,579	83,174	83,815	84,027	83,937	83,839	83,773
GC(%)	32.8	32.8	32.8	32.8	32.7	32.7	32.7	32.7	32.8	32.7	32.7
Length (%)	54.64	54.64	54.64	54.64	54.64	54.58	54.8	54.9	54.99	54.87	54.85
**SSC**											
Length (bp)	17,896	17,895	17,896	17,895	17,880	17,838	17,807	17,846	17,817	17,859	17,829
GC(%)	28.7	28.8	28.8	28.8	28.6	28.6	28.7	28.7	28.8	28.7	28.7
Length (%)	11.75	11.75	11.75	11.75	11.75	11.70	11.65	11.66	11.67	11.68	16.67
**IR**											
Length (bp)	25,574	25,574	25,574	25,574	25,530	25,485	25,591	25,575	25,432	25,542	25,563
GC(%)	41.8	41.9	41.9	41.9	41.9	41.9	41.9	41.9	41.8	41.9	41.9
Length (%)	16.80	16.8	16.8	16.8	16.77	16.72	16.74	16.71	16.66	16.71	16.73
**Total**											
GC(%)	35.4	35.4	35.4	35.4	35.3	35.3	35.3	35.3	35.3	35.3	35.3
Length (bp)	152,224	152,217	152,218	152,218	152,218	152,381	152,804	153,023	152,618	152,783	152,728

***G*.*soja*** ^***a***^ = *G*. *soja new* (in this study),

***G*.*soja*** = *G*. *soja* (old), ***G*.*max*** = *G*. *max*, ***G*.*graci*** = *G*.*gracilis*, ***G*.*canes*** = *G*.*canescens*, ***G*.*cyrtol*** = *G*. *cyrtoloba*, ***G*. *doli*** = *G*.*dolichocarpa*, ***G*. *falca*** = *G*. *falcata*, ***G*. *stenop*** = *G*.*stenophita*, ***G*.*syndet*** = *G*.*sydetika*, ***G*. *tome*** = *G*.*tomentella*

**Table 2:** Comparsion of coding and non-codign region size among ten *Glycine* species.

Region	*G*.*soja* ^*a*^	*G*.*soja*	*G*.*max*	*G*.*graci*	*G*.*canes*	*G*.*cyrtol*	*G*. *doli*	*G*. *falca*	*G*. *stenop*	*G*.*syndet*	*G*. *tome*
**Protein Coding**											
Length (bp)	79,250	77,835	77,769	77,811	77,607	72,294	77,649	77,598	77,646	77,604	77,601
GC(%)	36.2	36.1	36.1	36.1	36.1	36.8	36.1	36.1	36.1	36.1	36.1
Length (%)	52.06	51.13	51.12	51.11	50.98	47.44	50.91	50.71	50.8	50.7	50.8
**tRNA**											
Length (bp)	2,925	2,817	2,792	2,799	2,792	2,792	2,792	2,792	2,792	2,792	2,792
GC(%)	52.4	52.9	52.9	53.0	52.8	52.8	52.8	52.9	52.8	52.8	52.8
Length (%)	1.92	1.85	1.83	1.83	1.83	1.83	1.82	1.82	1.82	1.82	1.82
**rRNA**											
Length (bp)	9,054	9,054	9,054	9,054	9,054	9,054	9,054	9,054	9,054	9,054	9,054
GC(%)	54.9	54.9	54.9	54.9	54.9	54.9	54.9	54.9	54.9	54.9	54.9
Length (%)	5.94	5.94	5.94	5.94	5.94	5.94	5.93	5.91	5.93	5.93	5.94
**Intergenic**											
GC(%)	33.23	33.45	33.26	33.23	33.45	33.23	33.432	33.23	33.454	33.45	33.26
Length (bp)	60,995	62,511	62,603	62,554	62,765	68,241	63,309	63,579	63,123	63,333	63,281

***G*.*soja*** ^***a***^ = *G*. *soja* (in this study),

***G*.*soja*** = *G*. *soja* (old), ***G*.*max*** = *G*. *max*, ***G*.*graci*** = *G*.*gracilis*, ***G*.*canes*** = *G*.*canescens*, ***G*.*cyrtol*** = *G*. *cyrtoloba*, ***G*. *doli*** = *G*.*dolichocarpa*, ***G*. *falca*** = *G*. *falcata*, ***G*. *stenop*** = *G*.*stenophita*, ***G*.*syndet*** = *G*.*sydetika*, ***G*. *tome*** = *G*.*tomentella*

The total coding DNA sequences (CDSs) were 79,250 bp in length and encoded 87 genes, including 26,416 codons (Table 3). The codon-usage frequency of the *G*. *soja* plastome was determined based on tRNA and protein-coding gene sequences (Table 4). Leucine (10.6%) and cysteine (1.2%) were the most and least frequently encoded amino acids, respectively, and isoleucine, serine, glycine, arginine, and alanine constituted 9.0%, 7.7%, 6.5%, 5.8%, and 5.0% of the CDSs, respectively, as reported previously [54, 56].

**Table 3:** Base composition of the *G*. *soja* plastid genome.

	T/U(%)	C (%)	A (%)	G(%)	Length (bp)
**Genome**	32.3	17.4	32.4	18.0	152,224
**LSC**	33.6	16.0	33.6	16.8	83,181
**SSC**	35.3	13.6	36.0	15.1	17,896
**IR**	29.0	21.7	29.2	20.1	25,574
**tRNA**	25.2	23.1	22.4	29.3	2,925
**rRNA**	18.9	23.4	26.2	31.5	9,054
**Protein-coding genes**	32.2	17.0	31.5	19.2	79,250
**1st position**	24.1	18.3	31.6	25.8	26,416
**2nd position**	33.2	19.8	29.7	17.1	26,416
**3rd position**	39.2	12.7	33.2	14.7	26,416

**Table 4:** The codon-anticodon recognition pattern and codon usage for the *G*. *soja* plastid genome.

Amino acid	Codon	No	RSCU	tRNA	Amino acid	Codon	No	RSCU	tRNA
Phe	UUU	1099	1.28		Ala	GCA	395	1.18	*trnA-UGC*
Phe	UUC	503	0.7	*trnF-GAA*	Ala	GCG	122	0.5	
Leu	UUA	932	1.9	*trnL-UAA tRNA*	Tyr	UAU	846	1.5	
Leu	UUG	557	1.1	*trnL-CAA tRNA*	Tyr	UAC	165	0.47	*trnY-GUA tRNA*
Leu	CUU	589	1.29		Stop	UAG	1	0.74	
Leu	CUC	172	0.4		Stop	UGA	0	0.80	
Leu	CUA	381	0.87	*trnL-UAG tRNA*	Stop	UAA	5	1.44	
Leu	CUG	164	0.32		His	CAU	503	1.49	
Ile	AUU	1170	1.51		His	CAC	134	0.50	*trnH-GUG tRNA*
Ile	AUC	392	0.5	*trnI-GAU tRNA*	Gln	CAA	764	1.53	*trnQ-UUG tRNA*
Ile	AUA	827	0.89		Gln	CAG	200	0.49	
Met	AUG	499	1	*trnM-CAU tRNA*	Asn	AAU	1045	1.44	
Val	GUU	533	1.50		Asn	AAC	286	0.55	*trnQ-UUG tRNA*
Val	GUC	158	0.46	*trnV-GAC tRNA*	Lys	AAA	1181	1.44	*trnK-UUU tRNA*
Val	GUA	534	1.47	*trnV-UAC tRNA*	Lys	AAG	331	0.55	
Val	GUG	173	0.54		Asp	GAU	827	1.55	
Ser	UCU	591	1.56		Asp	GAC	204	0.44	*trnD-GUC tRNA*
Ser	UCC	298	1.23	*trnS-GGA tRNA*	Glu	GAA	1042	1.48	*trnE-UUC tRNA*
Ser	UCA	442	1.03	*trnS-UGA tRNA*	Glu	GAG	313	0.51	
Ser	UCG	181	0.48		Cys	UGU	231	1.50	
Ser	AGU	405	1.24		Cys	UGC	85	0.49	
Ser	AGC	120	0.42	*trnS-GCU tRNA*	Trp	UGG	442	1	*trnW-CCA tRNA*
Pro	CCU	403	1.59		Arg	CGU	339	1.36	*trnR-ACG tRNA*
Pro	CCC	202	0.86		Arg	CGC	91	0.51	
Pro	CCA	334	1.07	*trnP-UGG tRNA*	Arg	CGA	361	1.24	
Pro	CCG	122	0.47		Arg	CGG	100	0.48	
Thr	ACU	571	1.68		Arg	AGA	485	1.77	*trnR-UCU tRNA*
Thr	ACC	210	0.76	*trnT-GGU tRNA*	Arg	AGG	156	0.61	
Thr	ACA	421	1.08	*trnT-UGU tRNA*	Gly	GGU	585	1.28	
Thr	ACG	139	0.45		Gly	GGC	157	0.42	
Ala	GCU	623	1.72		Gly	GGA	691	1.52	*trnG-UCC tRNA*
Ala	GCC	189	0.59		Gly	GGG	282	0.77	

Among these codons, the most and least frequently used were AAA (n = 1,181), which encodes lysine, and ATC and ATT (n = 1, n = 1), which both encode methionine. The AT contents of the 1^st^, 2^nd^, and 3^rd^ codon positions of CDSs were 55.7%, 62.9%, and 72.4%, respectively (Table 3). The high AT content observed at the 3^rd^ codon position is similar to that reported for the plastomes of other terrestrial plants [54, 57, 58]. In addition, 46.36% and 57.65% of the preferred synonymous codons (RSCU > 1) ended with A or U and C or G, respectively, and 44.30% and 55.20% of the non-preferred synonymous codons (RSCU < 1) ended with C or G and A or U, respectively. However, there was no bias in start codon usage (AUG or UGG; RSCU = 1; Table 4).

The *G*. *soja* genome map (Fig 1) was representative of known *Glycine* plastomes in general, and no structural rearrangement was detected among these plastomes. The length of the *G*. *soja* plastome was 152,224 bp, which is similar to that of *G*. *max* (152,217 bp) [35], but smaller than those of *G*. *dolichocarpa*, *G*. *falcata*, *G*. *sydetika*, and *G*. *tomentella* (Table 1). Among the sequenced *Glycine* plastomes, that of *G*. *max* is smallest, and that of *G*. *dolichocarpa* is largest (Table 1). Furthermore, a total of 134 genes were identified in the *G*. *soja* plastome, of which 110 were unique, including 87 protein-coding genes, 39 tRNA genes, and eight rRNA genes (Fig 1, Table 5). Similar to other legumes, the plastome of *G*. *soja* lacked the *rpl22* gene, probably due to an ancient transfer to the nuclear genome [59]. The duplicated IR regions of the *G*. *soja* plastome resulted in complete duplication of the *rpl2*, *rpl23*, *ycf2*, *ycf15*, *ndhB*, and *rps7* genes as well as duplication of exons 1 and 2 of *rps12*, all four rRNA genes, and seven tRNA genes. The LSC region included 61 protein-coding and 24 tRNA genes, whereas the SSC region included only 12 protein-coding genes and one tRNA gene. The protein-coding genes included nine genes encoding large ribosomal proteins (*rpl2*, *14*, *16*, *20*, *22*, *23*, *32*, *33*, and *36*), 12 genes encoding small ribosomal proteins (*rps2*, *3*, *4*, *7*, *8*, *11*, *12*, *14*, *15*, *16*, *18*, and *19*), five genes encoding photosystem I components (*psaA*, *B*, *C*, *I*, and *J*), 16 genes related to photosystem II (Table 5), and six genes encoding ATP synthase and electron transport chain components (*atpA*, *B*, *E*, *F*, *H*, and *I*; Table 5).

**Table 5:** Genes in the sequenced *G*. *soja* chloroplast genome.

Category	Group of genes	Name of genes
**Self-replication**	Large subunit of ribosomal proteins	*rpl2*, *14*, *16*, *20*, *22*, *23*, *32*, *33*, *36*
	Small subunit of ribosomal proteins	*rps2*, *3*, *4*, *7*, *8*, *11*, *12*, *14*, *15*, *16*, *18*, *19*
	DNA dependent RNA polymerase	*rpoA*, *B*, *C1*, *C2*
	rRNA genes	*RNA*
	tRNA genes	*trnA-UGC*, *trnC-GCA*, *trnD-GUC*, *trnE-UUC trnF-GAA*, *trnfM-CAU*, *trnG-UCC*, *trnH-GUG*, *trnI-CAU*, *trnI-GAU*, *trnK-UUU*, *trnL-CAA*, *trnL-UAA*, *trnL-UAG*, *trnM-CAU*, *trnN-GUU*, *trnP-GGG*, *trnP-UGG*, *trnQ-UUG*, *trnR-ACG*, *trnR-UCU*, *trnS-GCU*, *trnS-GGA*, *trnS-UGA*, *trnT-GGU*, *trnT-UGU*, *trnV-GAC*, *trnV-UAC*, *trnW-CCA*, *trnY-GUA*
**Photosynthesis**	Photosystem I	*psaA*, *B*, *C*, *I*, *J*
	Photosystem II	*psbA*, *B*, *C*, *D*, *E*, *F*, *G*, *H*, *I*, *J*, *K*, *L*, *M*, *N*, *T*, *Z*
	NadH oxidoreductase	*ndhA*, *B*, *C*, *D*, *E*, *F*, *G*, *H*, *I*, *J*, *K*
	Cytochrome b6/f complex	*petA*, *B*, *D*, *G*, *L*, *N*
	ATP synthase	*atpA*, *B*, *E*, *F*, *H*, *I*
	Rubisco	*rbcL*
**Other genes**	Maturase	*matK*
	Protease	*clpP*
	Envelop membrane protein	*cemA*
	Subunit Acetyl- CoA-Carboxylate	*accD*
	c-type cytochrome synthesis gene	*ccsA*
**Unknown**	Conserved Open reading frames	*ycf1*,*2*, *3*, *15*

Among the coding genes, *rps12* was unequally divided, with its 5′ exon being located in the LSC region and one copy of the 3′ exon and intron being located in each of the IR regions, as in other angiosperms. The *ycf1* gene was located at the IRa/SSC boundary, leading to incomplete duplication of the gene within the IR regions. We also identified 12 intron-containing genes, including nine that contained a single intron and three (*ycf3*, *clpP*, and *rps12*) that contained two introns (Table 6). This is in contrast to the situation in *Cicer arietinum*, *Medicago truncatula*, *Trifolium subterraneum*, *Pisum sativum*, and *Lathyrus sativus*, all of which have lost an intron from both *clpP* and *rps12* [19]. The largest intron was found in *trnK-UUU* (2583 bp) and included the entire *matK* gene, whereas *trnL-UAA* contained the smallest intron (508 bp). Introns play an important role in the regulation of gene expression, and recent research has shown that introns can improve exogenous gene expression when located at specific positions. Therefore, introns can be a valuable tool for improving transformational efficiency [60]. Furthermore, intron sequences in legume chloroplast DNA have become important tools in phylogenetic analyses [61]. In addition, even though *ycf1* and *ycf2* [62, 63], *rpl23* [64], and *accD* [65, 66] are often absent in plants [64], they have been reported to occur the plastomes of various *Glycine* species [67]. *atpB-atpE* pairs were observed to overlap with each other by ~1 bp. However, *psbC-psbD* exhibited a 53-bp overlap in *G*. *soja* plastomes, similar to what is observed in *G*. *max* [35] and *G*. *falcata* [67], *Arabidopsis arenosa* (17-bp overlap) [68], *Gossypium* (53-bp overlap) [69], and *Camellia* (52-bp overlap) [70]. Previously, Addachi et al. (2012) [71] reported the importance of the partial overlap of *psbC* and *psbD* cistrons. They demonstrated that the translation of the *psbC* cistron largely depends on the translation of the preceding *psbD* cistron, indicating a contribution form independent *psbC* translation. Similar results were reported in tobacco, where *ndhC* and *ndhK* cistrons overlap, and *ndhK* translation is strictly dependent on the upstream termination codon [72].

**Table 6:** Length of exons and introns in intron-containing genes from the *Glycine soja* plastid genome.

Gene	Location	Exon I (bp)	Intron 1 (bp)	Exon II (bp)	Intron II (bp)	Exon III (bp)
*atpF*	LSC	144	736	414		
*clpP*	LSC	69	710	297	775	225
*ndhA*	SSC	552	1269	756		
*ndhB*^a^	IR	777	692	756		
*petB*	LSC	6	808	642		
*petD*	LSC	8	728	476		
*rpl2*^a^	IR	393	681	468		
*rpl16*	LSC	9	1165	402		
*rpoC1*	LSC	441	785	1638	719	159
rps12*		114	-	26	531	232
*rps16*	LSC	39	887	228		
*ycf3*	LSC	126	697	228	745	150
*trnA-UGC*	IR	38	810	35		
*trnI -GAU*	IR	42	948	35		
*trnL-UAA*	LSC	37	508	50		
*trnK -UUU*	LSC	37	2583	29		
*trnV-UAC*	LSC	39	586	37		

^*a*^ *replicated genes*

*The *rps12* coding sequence is split between *5′-rps12* and *3′-rps12*, which are located in the large single-copy region and inverted repeat region, respectively.

### Repeat sequence content

**Fig 2:**
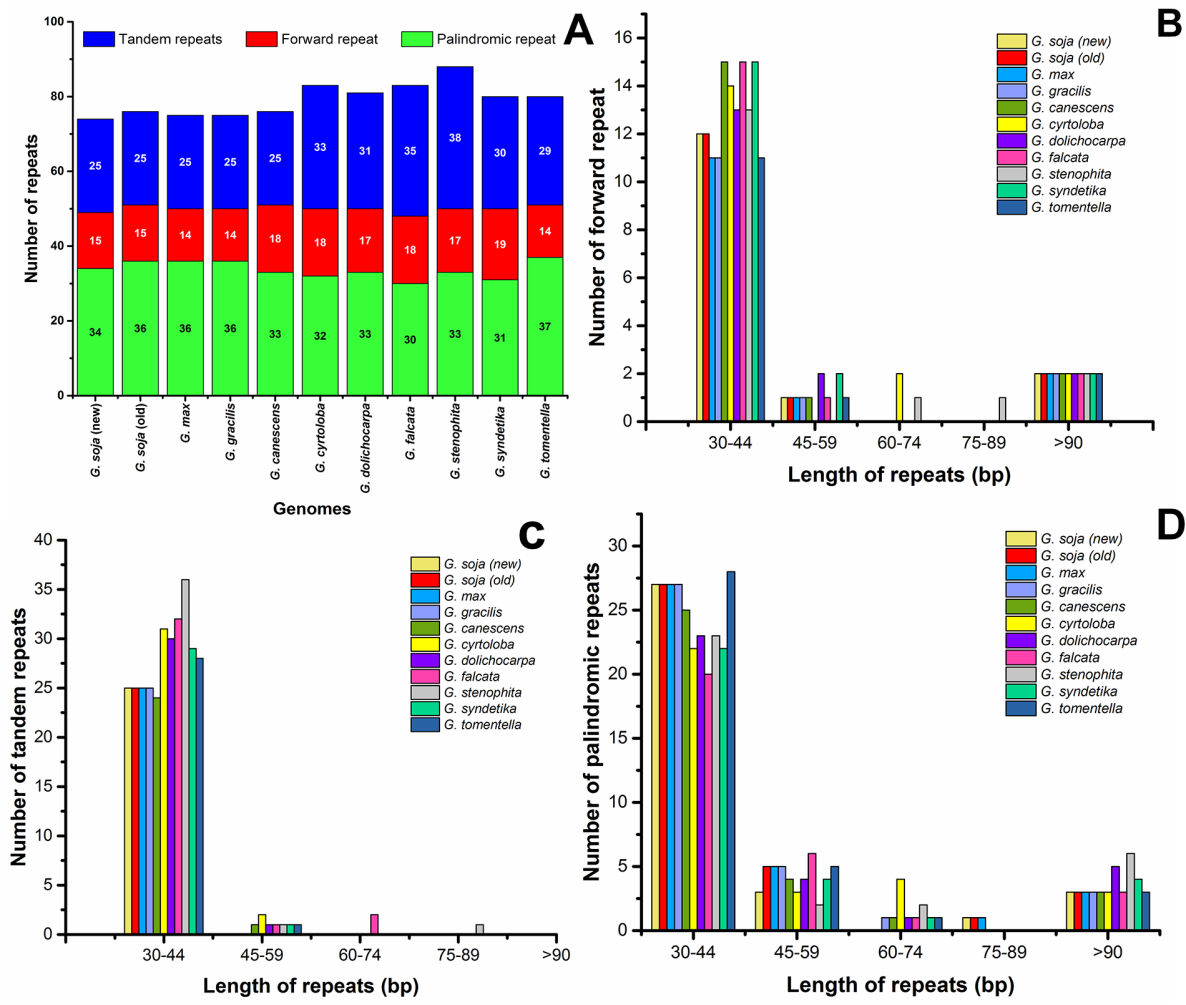
Analysis of repeated sequences in 10 *Glycine* plastid genomes. **A**, Total of three repeat types; **B**, Length distribution of forward repeat sequences; **C**, Length distribution of tandem repeat sequences; **D**, Length distribution of palindromic repeat sequences.

Repeat analysis of the *G*. *soja* plastome identified 34 palindromic repeats, 15 forward repeats, and 25 tandem repeats (Fig 2A). Among these repeats, 12 of the forward repeats were 30–44 bp in length, while all 25 tandem repeats were 15–29 bp in length (Fig 2A–2D). Similarly, 27 of the palindromic repeats were 30–44 bp in length, and three repeats were 45–59 bp in length (Fig 2D). Overall, 74 repeats were identified in the *G*. *soja* plastome, which is a similar number to the 75, 75, 76, 83, 81, 83, 88, 80, and 80 repeat sequences found in the plastomes of *G*. *max*, *G*. *gracilis*, *G*. *canescens*, *G*. *cyrtoloba*, *G*. *dolichocarpa*, *G*. *falcata*, *G*. *stenophita*, *G*. *syndetika*, and *G*. *tomentella*, respectively (Fig 2A). Therefore, *G*. *soja* is more similar to *G*. *max* and *G*. *gracilis* in terms of repeats. Approximately 29.4% of these repeats were distributed in protein-coding regions. Previous reports suggest that repeat sequences, which contribute to genome rearrangements, can be very helpful in phylogenetic studies [58, 73]. In addition, analyses of various plastomes have shown that repeat sequences induce indels and substitutions [74], and both sequence variation and genome rearrangement occur as a result of slipped-strand mispairing and improper recombination of such repeat sequences [73, 75, 76]. Furthermore, the presence of repeat sequences indicates that loci are hotspots for genome reconfiguration [58, 77], and repeats can be used to develop genetic markers for phylogenetic and population studies [58].

### SSR content

**Fig 3:**
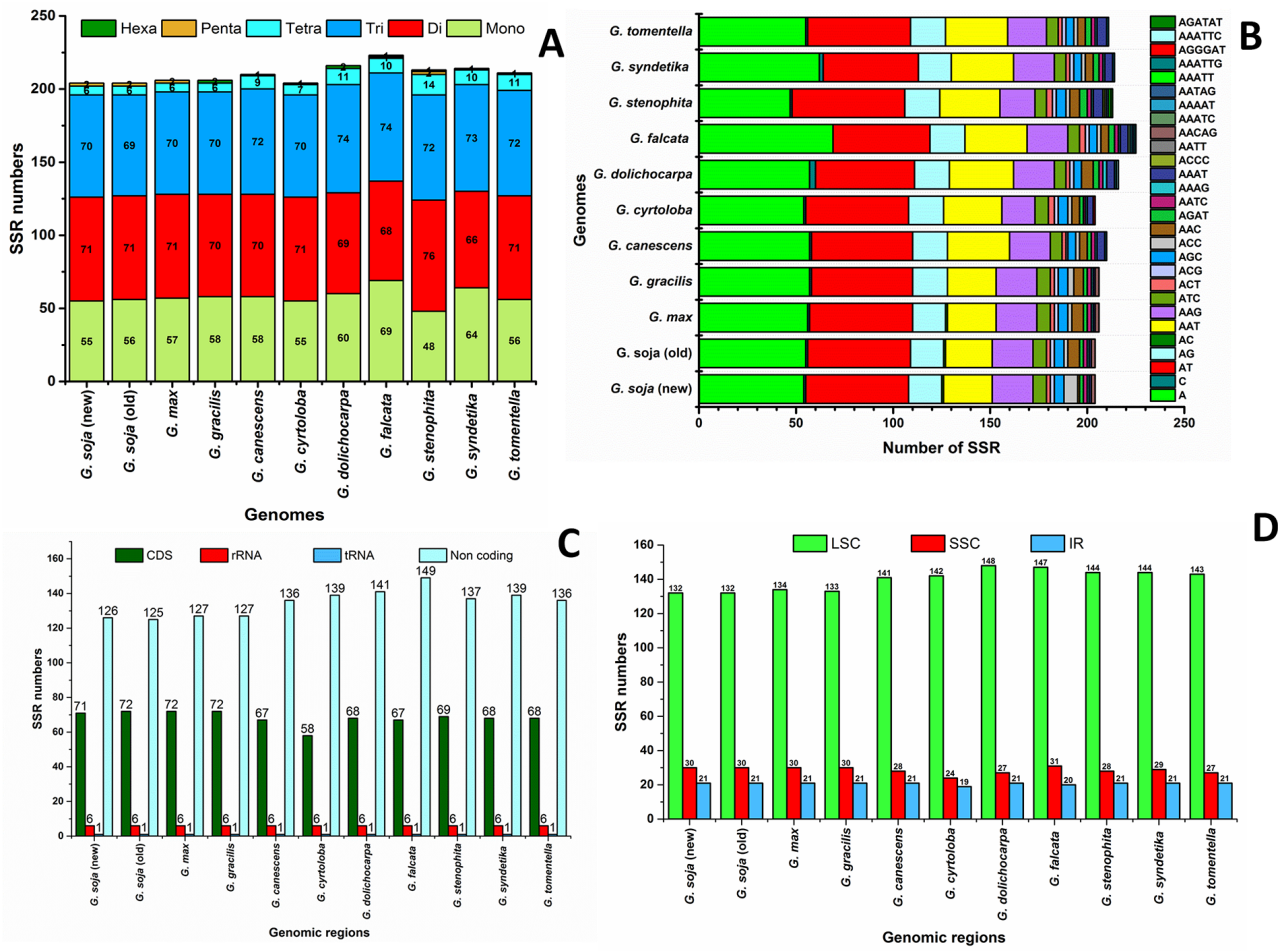
Analysis of simple sequence repeats (SSRs) in the ten *Glycine* plastid genomes. **A**, Number of SSR types; **B**, Frequency of identified SSR motifs in different repeat class types; **C**, Frequency of identified SSRs in coding regions; **D**, Frequency of identified SSRs in the small single-copy (SSC), large simple-copy (LSC), and inverted repeat (IR) regions.

Simple sequence repeats (SSRs), or microsatellites, are repeating sequences, typically of 1–6 bp in length, that are distributed throughout the genome. In the present study, we identified perfect SSRs in the plastome of *G*. *soja* and in those of nine other *Glycine* species (Fig 3A). Certain parameters were set because SSRs of 10 bp or longer are prone to slipped-strand mispairing, which is believed to be the main mechanism of the formation of SSR polymorphisms [78–80].

A total of 204 perfect microsatellites were identified in the *G*. *soja* plastome (Fig 3A), which is a similar number to the 206, 206, 210, 204, 216, 223, 213, 214, and 211 perfect microsatellites identified in the plastomes of *G*. *max*, *G*. *gracilis*, *G*. *canescens*, *G*. *cyrtoloba*, *G*. *dolichocarpa*, *G*. *falcata*, *G*. *stenophita*, *G*. *syndetika*, and *G*. *tomentella*, respectively (Fig 3A). The majority of the SSRs possessed dinucleotide repeat motifs, varying in number from 66 in *G*. *soja* to 76 in *G*. *falcata* and *G*. *dolichocarpa*, while trinucleotide SSRs were the second most common, ranging in number from 69 in *G*. *syndetika* to 74 in *G*. *stenophita*. Using our search criterion, two pentanucleotide SSRs were identified in *G*. *soja*, *G*. *max*, and *G*. *stenophita*, and two hexanucleotide SSRs were identified in *G*. *gracilis* and *G*. *dolichocarpa* (Fig 3A). In *G*. *soja*, the majority of the mononucleotide SSRs were A (98.1%) and C (1.81%) motifs, and the majority dinucleotide SSRs were A/T (71.64%) and A/G (23.940%) motifs (Fig 3B, Table 7). In addition, 61.7% of the SSRs were located in non-coding regions, whereas 2.9% and 0.49% were located in rRNA and tRNA genes, respectively (Fig 3C). Further analysis indicated that 64.7% of the SSRs were located in the LSC region, whereas 20.58% and 14.7% were located in the IR and SSC regions, respectively (Fig 3D). These results are similar to previous reports that SSRs are unevenly distributed in plastomes, and the findings might provide more information for selecting effective molecular markers for detecting intra- and interspecific polymorphisms [81–84]. Furthermore, most of the mono- and dinucleotide repeats consisted of A and T, which may have contributed to the bias in base composition, as in the plastomes of other species [85]. Our findings are comparable to previous reports that SSRs in plastomes are generally composed of polythymine (polyT) or polyadenine (polyA) repeats and infrequently contain tandem cytosine (C) or guanine (G) repeats [86], thereby contributing to AT richness [55, 56, 86].

**Table 7:** Simple sequence repeats (SSRs) in the *Glycine soja* plastid genome.

Unit	Length	No.	SSR start
**A**	18	1	51,531
	16	2	92,627, 142,764
	15	2	76,538, 119,451
	14	2	33,433, 82,862
	13	4	24,610, 51701, 110,244, 111,377
	12	7	6,968, 9,644, 9,656, 58,365, 62,260, 75,661, 82,660
	11	15	14,313, 42,712, 54,965, 59,329, 70698, 78,955, 79,488, 81,034, 81,302, 10,9835, 111,046, 111,519, 111,927, 112,225, 122,146
	10	22	2,991, 4,452, 7,568, 25,542, 31,495, 34,893, 38,160, 38,510, 45,234, 46,902, 54,259, 56,682, 62,419, 66,716, 67,450, 69,278, 93297, 109,698, 110,547, 114,419, 124,220, 142,100
**C**	12	1	9644
**AT**	19	1	5,177
	17	1	5,159
	16	1	24,676
	14	1	32,841
	13	1	48,415
	12	2	54,297, 118,666
	11	8	33,695, 48,440, 65,081, 67,502, 68,320, 78,342, 79,508, 122,331
	10	5	31,746, 32,806, 68,072, 80,714, 116,632
	9	9	13,837, 35,671, 54,930, 58,400, 60,678, 64,792, 69,490, 82,699, 120,175
	8	24	100, 1,607, 2,068, 3,635, 4,513, 4,526, 13,370, 16,835, 28,206, 47,399, 51,596, 51,773, 51,795, 58,249, 60,155, 65,092, 69,374, 76,625, 79,531, 82,378, 92,346, 116,291, 123,690, 143,053
**AG**	9	2	25,492, 28,221
	8	15	3,673, 6,261, 85,791, 86,793, 94,040, 105,226, 105,546, 107,047, 120,875, 128,352, 129,853, 130,173, 141,359,148,606, 149,608
**AC**	9	1	120,511
**AAT**	15	1	28,637
	13	1	14,614
	12	1	29,635
	11	1	73,972
	10	6	2,980, 14,647, 23,469, 47,482, 61,211, 83,153
	9	15	4,840, 6,885, 18,582, 24,528, 28,614, 32,259, 32,318, 45,719, 47,151, 58,337, 80,973, 99,425, 115,619, 120,102, 135,973
**AAG**	12	1	2,123
	11	1	111,544
	10	4	83,359, 95,785, 139,612, 152,038
	9	15	23,601, 39,016, 61,479, 69,713, 76,888, 89,691, 91,515, 94,335, 102,444, 109,943, 117,624, 133,154, 141,063, 143,883, 145,707
**ATC**	11	1	57,126
	9	6	22,369, 40,828, 45,626, 83,824, 116,434, 151,574
**ACG**	10	2	83,313, 152,084
**AGC**	9	5	5,366, 20,175, 68,568, 103,665, 131,733
**ACC**	9	2	58,920, 90,061
**ACT**	9	1	66,702
**AGAT**	15	2	18,423, 18,450
**AATC**	13	1	119,923
	12	1	78,291
**AAAG**	12	1	67,682
**AAAT**	12	1	117,190
**AACAG**	15	2	107,707, 127,685

### Sequence and structural divergence of *Glycine* plastid genomes

Ten complete *Glycine* plastomes were compared with the *G*. *soja* plastome. Analysis of genes with known functions indicated that *G*. *soja* shared 76 protein-coding genes with nine *Glycine* species. In addition, the gene content and organization of the *G*. *soja* plastome were similar to those of other *Glyine* species plastomes [67], but different from the usual gene order of angiosperm plastomes, due to a large inversion (~51 kb) that reversed the order of the genes between *trnK* and *accD* (Fig 1). This 51-kb inversion was previously reported in other members of the legume family, especially members of subfamily Papilionaoideae [16, 24, 87], and other inversions have been reported in the plastomes of other species, including a 5.6-kb inversion in *Milletia* [88], a 78-kb inversion in various closely related legumes, including *Phaseolus* and *Vigna* [17, 89], and a 36-kb inversion within the 51-kb inversion found in *Lupinus* and other genisotoids [90]. This change in gene order has been ascribed to the contraction and expansion of IR regions, leaving the gene order as described in papilionoids, retaining the 51-kb inversion, but alerting the genes bordering the IR region [89, 91].

Furthermore, the IR region overlaps the *ycf1* gene by 478 bp, as observed in legumes exhibiting the same inverted repeat as *G*. *soja*. This feature has been shown to distinguish the plastomes of legumes from those of other angiosperms, in which the IR region and *ycf1* typically overlap by 1,000 bp [35]. Moreover, as found in the plastomes of other legumes, the plastome of *G*. *soja* possessed variation and was missing two cp genes, *rpl22* and *infA*, [18], both of which have been replaced by cp-targeting nuclear copies [59, 92]. Absence of the *rps16* gene from the plastome has also been reported in other legume lineages, excluding *Glycine*, and the mitochondrial copy is dually targeted to both the cp and mitochondria [19, 93]. Furthermore, loss of the introns in *rps12* and *clpP* has been detected in the plastomes of various species [19], including those of *Glycine* species [35, 67].

**Fig 4:**
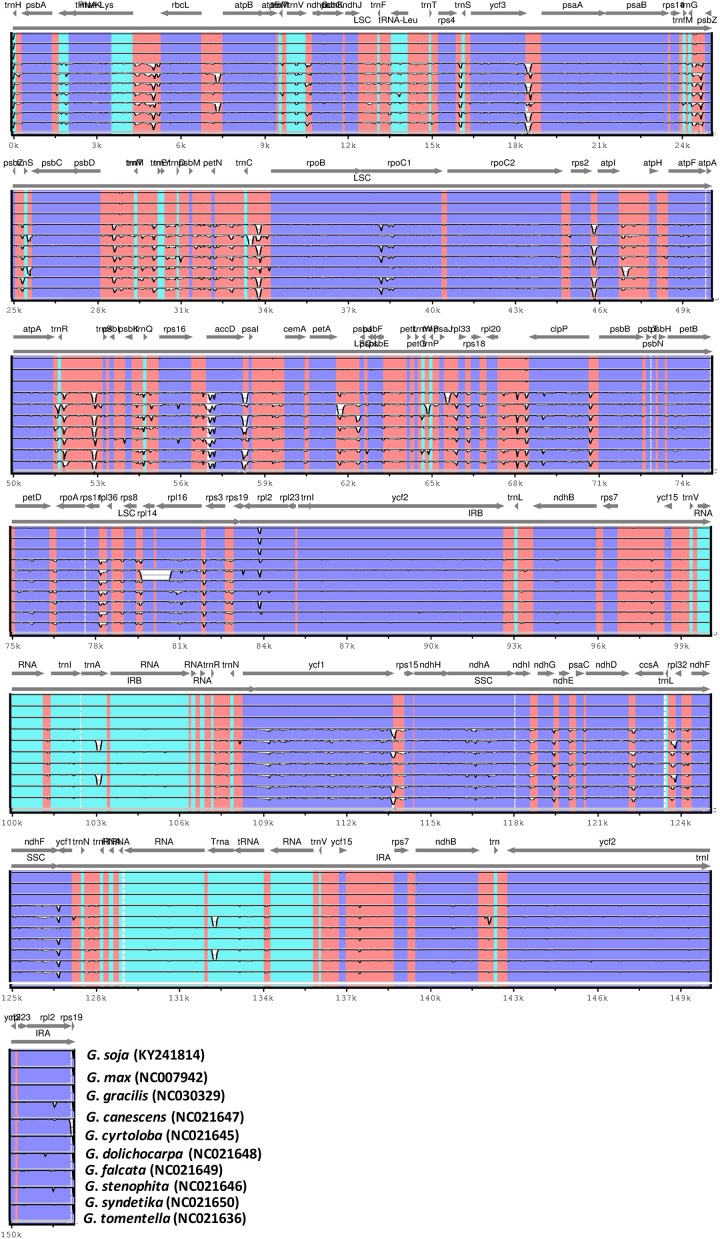
Visual alignment of plastid genomes from *Glycine soja* (new and old) and nine other *Glycine* species. VISTA-based identity plot showing the sequence identity among the ten *Glycine* species, using *G*. *soja* (new) as a reference. Vertical scale indicates the percentage of identity, ranging from 50% to 100%. Horizontal axis indicates the coordinates within the chloroplast genome. Arrows indicate the annotated genes and their transcriptional direction. A thick black line indicates the inverted repeat (IR) regions.

Pairwise alignment of the new *G*. *soja* plastome with the old *G*. *soja* plastome and those of nine other genomes showed a high degree of synteny. The annotation of the new *G*. *soja* plastome was used as a reference for plotting the overall sequence identity of the plastomes of the other ten *Glycine* species in mVISTA (Fig 4). In the results, relatively lower sequence identity was observed between the plastomes of the seven other perennial species, especially in the *rpoC1*, *atpF*, *accD*, *clpP*, *rpl2*, *ndhA*, *ndhF*, *rps8*, *rps19*, and *ycf1* genes (Fig 4). In addition, the LSC and SSC regions were less similar than the two IR regions in all *Glycine* species, and the non-coding regions were more divergent than the coding regions. Highly divergent regions included the *matK*-*rbcL*, *ycf3-psaA*, *trnC-rpoB*, *rpl20-clpP*, *rps16-trnQ*, *trnfM-trnM*, *psbM-petN*, *atpI-atpH*, *petA-psbJ*, and *ycf1-rps15* spacers, as reported previously [54, 55]. Our results also confirmed similar differences among various coding regions in the analysed species, as suggested by Kumar et al. [94]. On the other hand, *G*. *soja* exhibited high sequence identity with annual *Glycine* species (S1 Fig), which suggest that they are highly conserved. However, the variation in similarity levels revealed various coding and non-coding regions where the *G*. *soja* exhibits divergence from these annual *Glycine* species (S1 Fig). Similarly, we detected 10 relatively highly variable regions, including 4 gene regions and 6 intergenic regions of the cp genomes, that might be undergoing more rapid nucleotide substitution at species and cultivar levels (S2 Fig) (*atpB-rbcL*, *trnT-trnL*, *trnS(GGA)*-*trnG(UCC)*, *psbD-trnT*, *rps16*, *rpl33-rpl18*, *rpl16-rps3*, *ndhB*, *ycf1* and *ycf15*). These regions can be used as potential molecular markers for application in phylogenetic analyses of *Glycine*. Furthermore, various researchers have determined coding and non-coding regions of particularly high variability as potential molecular markers for *Glycine* species, such as *trnS(GGA*)-*trnG(UCC)*, *rpl16-rps3*, *trnT-trnL* and *atpB-rbcL* [95–97]. Similarly, it has been reported that non-coding regions in cp DNA show greater variability in nucleotide regions than coding regions, and these regions have become a major source of variability for phylogenetic studies in various species, including studies within *Glycine* species [98–100]. Furthermore, comparison of the plastomes of *G*. *soja* and related species revealed 72 SNPs and 26 indels in relation to *G*. *max* and *G*. *gracilis*, respectively (S2 Table). These results confirmed that the highly conserved plastome can include interspecific mutations that may be useful for analysing both genetic diversity and phylogenetic relationships.

**Fig 5:**
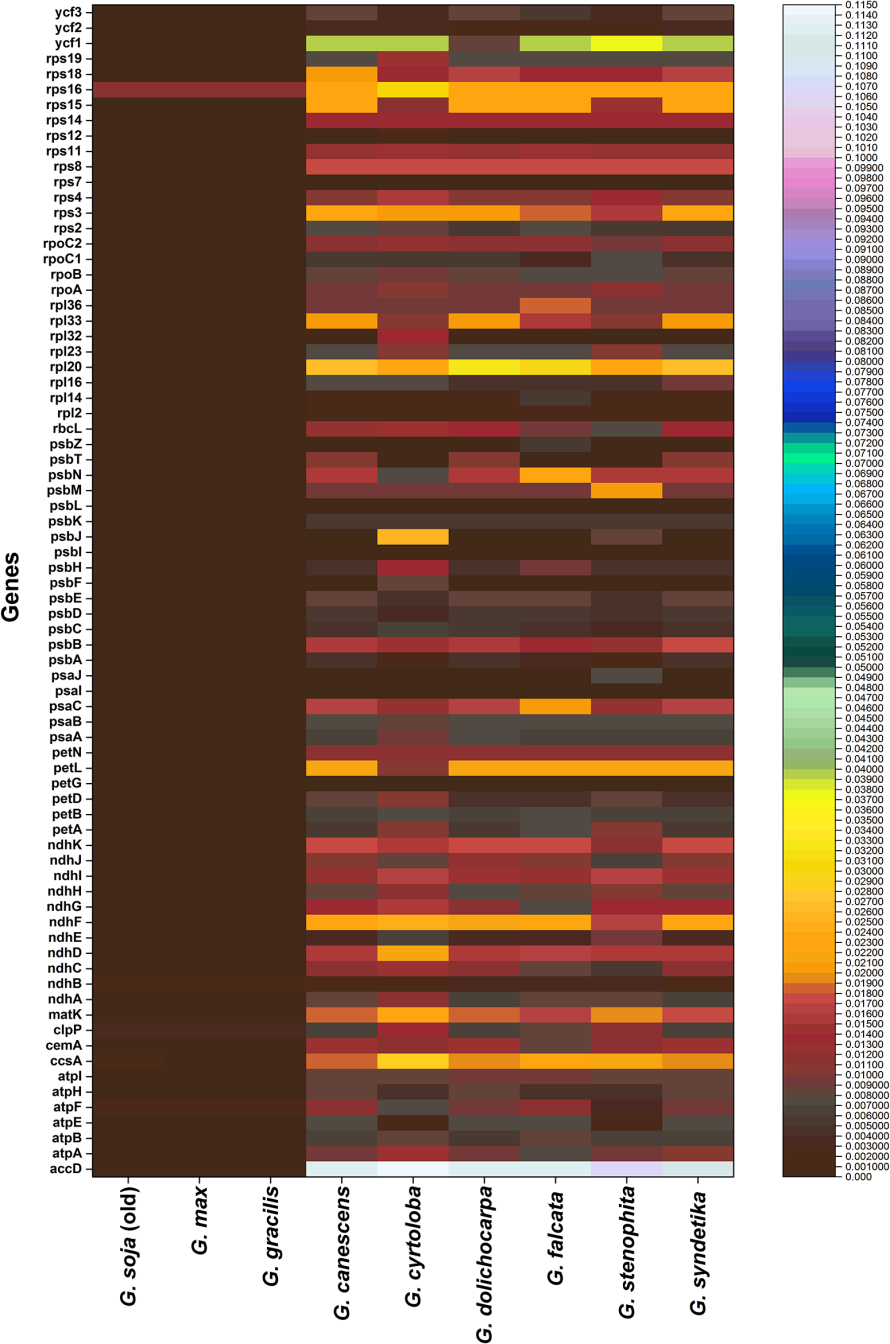
Pairwise distance of 76 genes from *Glycine soja* (new and old) and nine other *Glycine* species.

Similarly, we calculated the average pairwise sequence divergence among the plastomes of the ten *Glycine* species (S3 Table). The plastome of *G*. *soja* exhibited an average sequence divergence of 0.0096, whereas that of *G*. *cyrtoloba* possessed the highest average sequence divergence (0.00567), and those of *G*. *soja* and *G*. *max* displayed the lowest average sequence divergence (0.00010 and 0.00020, respectively). Furthermore, the nine most divergent genes among these genomes were *accD*, *matK*, *ycf1*, *rps16*, *rpl20*, *psbM*, *psbN*, *petL*, and *petN*. The *accD* gene exhibited the greatest average sequence divergence (0.07825), followed by *ycf1* (0.0241), *rps16* (0.0201), and *matK* (0.0194; Fig 5), most of which were located in the LSC region, and the *accD* gene of *G*. *soja* was highly divergent from those of nine other *Glycine* species (S3 Fig). The highest nucleotide diversity (Pi) (0.0916) and total number of mutations (Eta) (119 bp) in comparison with the *G*. *soja accD* gene was observed in *G*. *cyrtoloba* among the plastomes of the nine *Glycine* species, whereas the lowest were observed in *G*. *syndetika* (S4 Table). The length of the *accD* gene was 1,299 bp (433 aa) in *G*. *soja*, *G*. *max*, and *G*. *gracilis* and 1527 bp (523 aa) in the seven other *Glycine* species (S3 Fig). Similar differences in gene length within small cpDNA regions have been observed in a variety of other angiosperms [21]. In legume species, both *ycf4* and *accD* exhibit extensive length variation. The expansion of the *accD* gene is partly explained by the presence of numerous tandemly repeated sequences [21]. This *accD* gene encodes a subunit of acetyl-CoA carboxylase, which is related to fatty acid synthesis within the plastid. Previous gene knockout experiments have shown that the function of *accD* is vital, and this gene is expected to be indispensable [101]. However, various studies have identified widespread pseudogenization or absence of *accD* in a variety of relatively distant lineages, including the Ericaceae, Campanulaceae, Geraniaceae, Acoraceae, Poaceae, and Fabaceae [10, 21, 102–106], which implies that deletion or pseudogenization events occur independently.

### Boundaries between single-copy and IR regions

**Fig 6:**
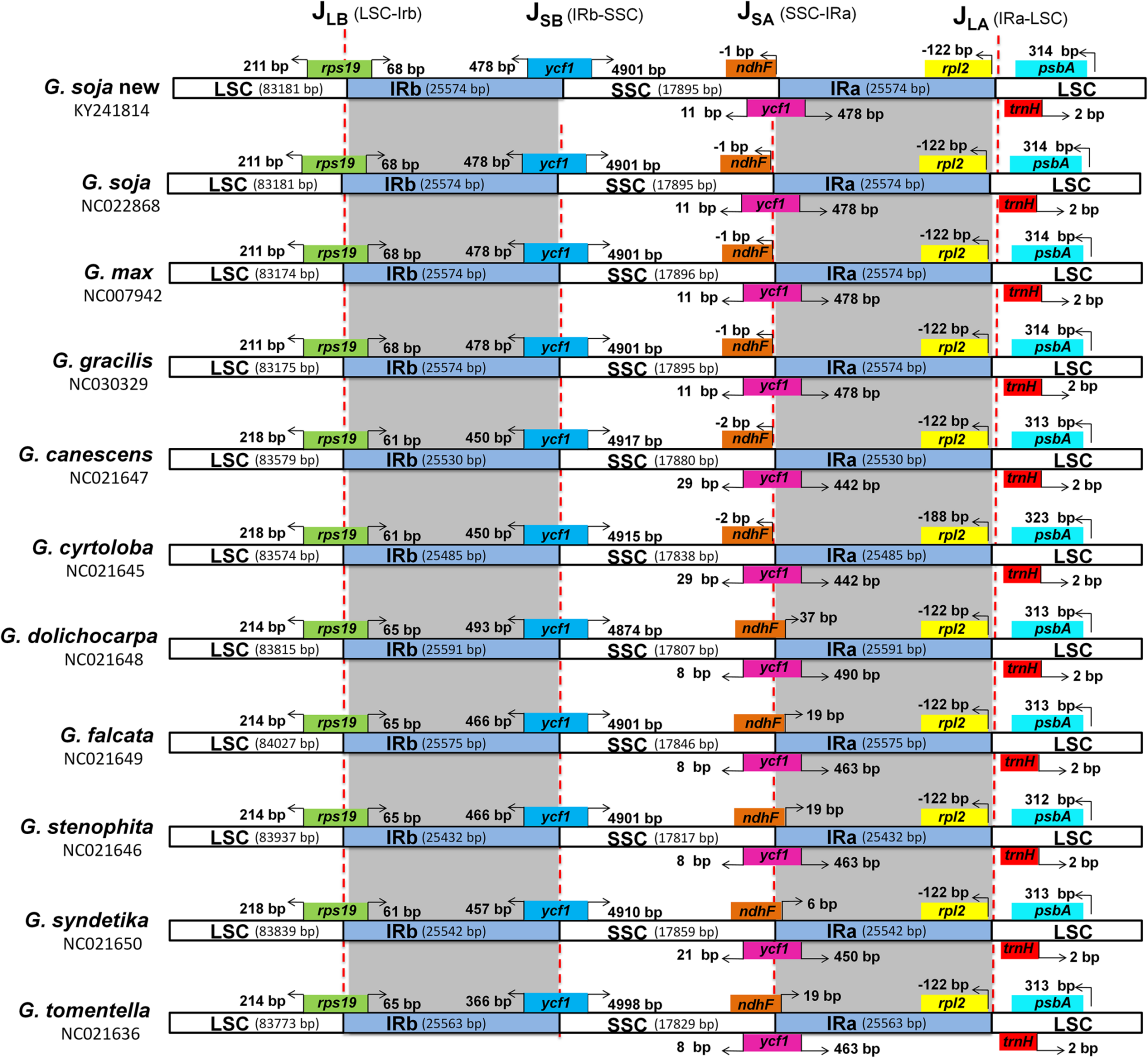
Distance between adjacent genes and junctions of the small single-copy (SSC), large single-copy (LSC), and two inverted repeat (IR) regions of the plastid genomes from ten *Glycine* species. Boxes above and below the main line indicate the adjacent bordering genes. The figure is not to scale in regard to sequence length and only shows relative changes at or near the IR/SC borders.

Variations in the size of angiosperm plastomes are mostly the result of expansion or contraction of the IR regions [79, 107–109]. In the present study, a detailed comparison of the four junctions (J_LA_, J_LB_, J_SA_, and J_SB_) between the two IR regions (IRa and IRb) and the two single-copy regions (LSC and SSC) of the 10 *Glycine* species was performed (Fig 6). Despite the similar lengths of the IR regions of *G*. *soja* and the other nine *Glycine* species, some expansion and contraction were observed, with the IR regions ranging from 25,432 bp in *G*. *stenophita* to 25,591 bp in *G*. *dolichocarpa*. The genes that marked the beginnings and ends of the IR regions were only partially duplicated, including 68 bp of *rpp19* in *G*. *soja*, *G*. *max*, and *G*. *gracilis* and 65 bp of *rpp19* in *G*. *dolichocarpa*, *G*. *falcata*, *G*. *stenophita*, and *G*. *tomentella*. In *G*. *canescens*, *G*. *syndetika*, and *G*. *cyrtoloba*, this distance was 61 bp in IR region from J_LB_. Similarly, the hypothetical cp gene *ycf1* was partially duplicated, with 478 bp of this sequence being duplicated in *G*. *soja*, *G*. *max*, and *G*. *gracilis*; 463 bp in *G*. *falcata*, *G*. *stenophita*, and *G*. *tomentella*; and 442 bp in *G*. *canescens* and *G*. *cyrtoloba*. J_LA_ was located between *rpl2* and *psbA*, and the distance between *rpl2* and J_LA_ was 122 bp in all of the species except for *G*. *cyrtoloba*, where *rpl2* is located 188 bp from the J_LA_ border. Additionally, the distance between *psbA* and the J_LA_ in the *G*. *soja* plastome was 314 bp, which was similar to that in the *G*. *max* and *G*. *gracilis* plastomes. Furthermore, the *ndhF* gene traversed the SSC and IRa regions, with 1 bp being located in the IR region of *G*. *soja*, 37 bp being located in the IR region of *G*. *dolichocarpa*, and 19 bp being located in the IR region of *G*. *falcata* and *G*. *stenophita* (Table 7).

### Phylogenetic relationships among *Glycine* species

**Fig 7:**
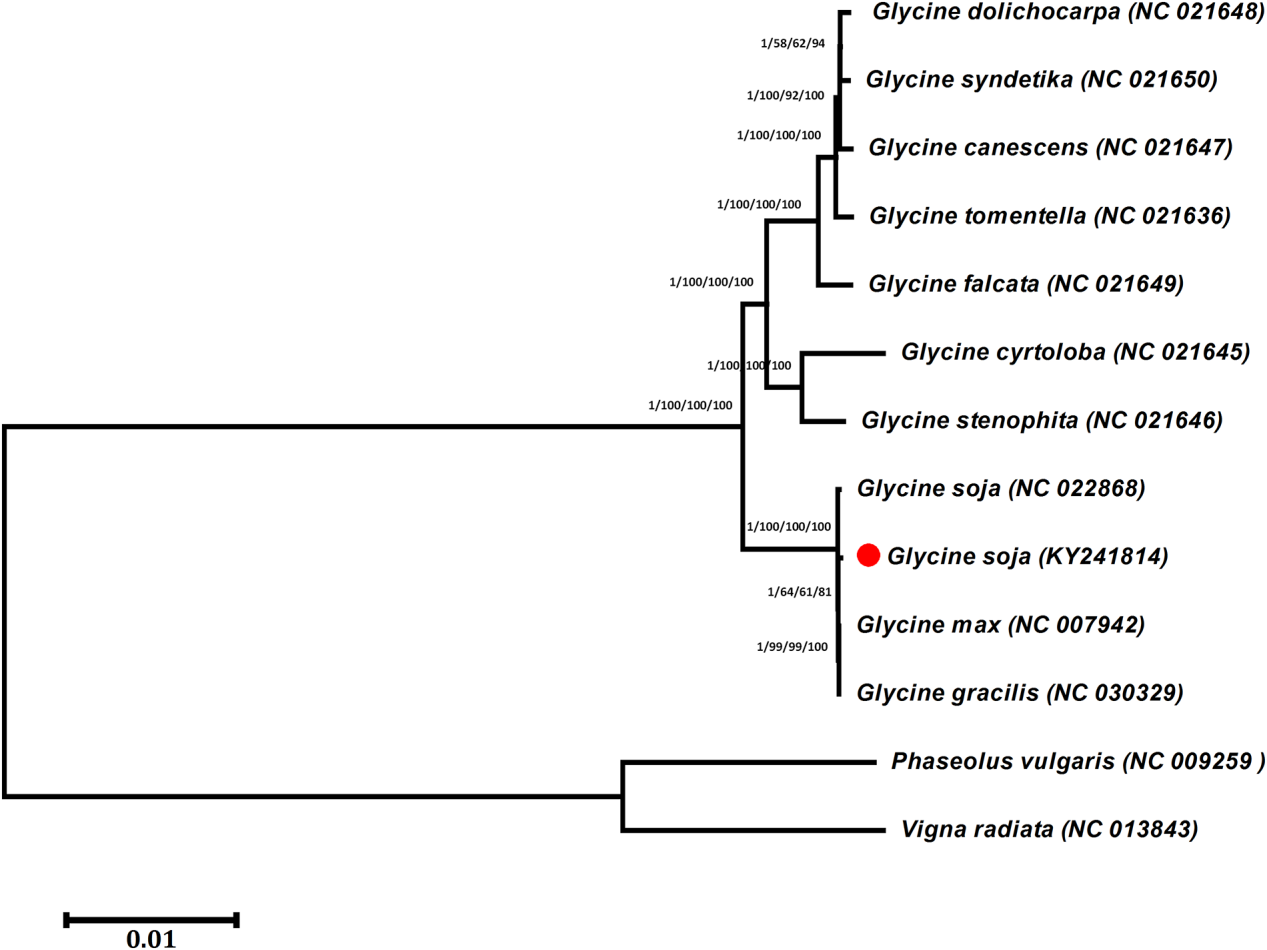
Phylogenetic trees of ten *Glycine* species. The whole-genome dataset was analysed using four different methods: neighbour-joining (NJ), maximum parsimony (MP), maximum likelihood (ML), and Bayesian inference (BI). Numbers above the branches represent bootstrap values in the NJ, MP, and ML trees and posterior probabilities in the BI trees. A red dot represents the position of *G*. *soja* (KY241814).

Plastid genomes have been useful in phylogenetic, evolutionary, and molecular studies. During the last decade, many analyses based on the comparison of plastid protein-coding genes [110, 111] and complete genome sequences [112] have addressed phylogenetic questions at deep nodes and enhanced our understanding of enigmatic evolutionary relationships among angiosperms. The genus *Glycine* includes 28 species, separated into two subgenera (*Soja* and *Glycine*), the former of which includes both cultivated soybean (*G*. *max*) and its wild annual progenitor (*G*. *soja*), which are distributed in East Asia, including Japan, Korea, China, Russia, and Taiwan. *G*. *max* and *G*. *soja* are both diploid (2n = 40) and interfertile and are thought to share highly similar genetic variation, although *G*. *soja* is much more variable than *G*. *max* [25, 113]. Polymorphisms in the cpDNA of *G*. *max* and *G*. *soja* have been used in numerous studies to assess maternal lineages and cytoplasmic diversity [114–119]. Continued efforts have expanded our ability to differentiate and understand the genomic structure and phylogenetic relationships of *Glycine* species [28, 120, 121]. The phylogeny and taxonomy of *Glycine* species in the *Soja* subgenus have been extensively investigated based on DNA variation, including nucleotide variation in nuclear ribosomal DNA (rDNA), intergenic spacer (ITS) regions [122], cpDNA restriction sites [24, 29], the histone gene H3-D [31], A-199a [123], and cpDNA intergenic spacer regions [25]. However, the complete genome sequence provides more detailed insight [52, 55, 124]. In the present study, the phylogenetic position of *G*. *soja* within its genus was established using the complete plastomes (S5 Table) and shared genes of 10 *Glycine* species and various methods of phylogenetic analysis. Phylogenetic analysis indicated that the complete plastome and the 76 shared genes contained the same phylogenetic signal. In both datasets, *G*. *soja* formed a clade with *G*. *max* and *G*. *gracilis*, with high BI and bootstrap support values (Fig 7, S4 Fig). Moreover, the tree topology confirmed previously reported relationships based on SSR and plastome data [114, 125]. These results of the present study are in general agreement with the results of Gao and Gao (2016) [37], who reported that *G*. *gracilis* is intermediate between the two species and is more closely related to *G*. *max* than *G*. *soja*. Furthermore, the results of the present study suggest that there is no conflict between the complete genome and the 76 shared gene datasets.

## Conclusions

In the present study, the complete plastome sequence of *G*. *soja* (152,224 bp) was determined. The gene order and structure of the *G*. *soja* plastome were found to be highly conserved with the plastomes of other *Glycine* species. The present study also revealed the distribution and location of repeat sequences and SSRs as well as the sequence divergence among the plastomes and shared genes between *G*. *soja* and nine of its congeners. No major structural rearrangement was observed in relation to annual *Glycine* species. However, in the perennial species, *accD* was found to be the most divergent gene, while relatively lower identity was observed in some other regions, especially in the *rpoC1*, *atpF*, *accD*, and *clpP* genes. Furthermore, phylogenetic analyses based on complete plastomes and shared genes yielded trees with the same topology, at least in regard to the placement of *G*. *soja*. Thus, the present study provides a valuable analysis of the complete plastome of *G*. *soja* and related species, which may facilitate species identification and both biological and phylogenetic studies.

## Supporting information

S1 Table Primers used for gap closing and sequence verification in *Glycine soja*. (DOCX)

S2 Table Indel and SNP analysis of the plastid genomes of *Glycine soja* (new and old) and nine other *Glycine* species. (XLSX)

S3 Table Average pairwise distance of plastid sequences from *Glycine soja* (new and old) and nine other *Glycine* species. (XLS)

S4 Table Comparison of the nucleotide variability (Pi) and total number of mutations of the *G*. *soja* accD gene with related species. (XLSX)

S5 Table Alignment of complete plastomes from *Glycine soja* (new and old) and 9 other *Glycine* species (NEXUS format). (ZIP)

S1 Fig Visual alignment of plastid genomes from *Glycine soja* (new) with annual *Glycine* speices (*G*. *soja* (old), *G*. *max* and *G*. *gracilis*). VISTA-based identity plot showing the sequence identity among the ten *Glycine* species, using *G*. *soja* (new) as a reference. Vertical scale indicates the percentage of identity, ranging from 70% to 100%. Horizontal axis indicates the coordinates within the chloroplast genome. Arrows indicate the annotated genes and their transcriptional direction. A thick black line indicates the inverted repeat (IR) regions.
(TIF)

S2 Fig Sliding window analysis of the complete plastome from *Glycine soja* (new) with annual *Glycine* speices (*G*. soja old, *G*. *max* and *G*. *gracilis*) (Window length: 800 bp, step size: 200 bp). X-axis, position of the midpoint of a window; Y- axis, nucleotide diversity of each window.
(TIF)

S3 Fig Alignment of *accD* gene nucleotide sequences among 11 *Glycine* species plastomes. (JPG)

S4 Fig Phylogenetic trees were constructed for ten species from the *Glycine* genus using different methods, and the Bayesian tree for the whole-genome sequences is shown. The data from the 76 shared genes were analysed with four different methods: joining-joining (NJ), maximum parsimony (MP), maximum likelihood (ML) and Bayesian inference (BI)). The numbers above the branches are the bootstrap values from the NJ, MP, and ML methods and the posterior probabilities of BI. A red dot represents the position of *G*. *soja* (KY241814).
(TIF)
