# Supplementary material for: Comparative analysis of complete plastid genomes from wild soybean (Glycine soja) and nine other Glycine species
Source: PLoS One. 2017 Aug 1;12(8):e0182281. doi: 10.1371/journal.pone.0182281 (PMC5538705; doi:10.1371/journal.pone.0182281)
Supplement: S1 Table — (DOCX) [file pone.0182281.s001.docx]

| Number | Primers | Sequence | Size | Information |
| --- | --- | --- | --- | --- |
| 1 | 23494-F | TCCTTCTATGGGGAAGGAAG | 725 bp | Gap Closing |
|  | 24198-R | ATTTAATAGTCATAGTAAGAA |  |  |
| 2 | 65974-F | ATGGCCAAAGGTAAAGATATCCGA | 586bp | Gap Closing |
|  | 66536-R | TTTCCTTGTTCACTAATAAATCGA |  |  |
| 3 | 86067-F | GACAAGGCTTCTTACTATACCTAT | 616bp | Gap Closing |
|  | 86659-R | AGCCCGCTTGTTTCTCAAGAAGAG |  |  |
| 4 | 108752-F | CGATGCAACAATAAATTTATATTT | 782bp | Gap Closing |
|  | 109510-R | TCCATTACTTTTGCCCTATTTACA |  |  |

**S1 Table. Primers used for gap closing and sequencing verification in *G. soja***
