# Supplementary figures and images for: Comparative analysis of complete plastid genomes from wild soybean (Glycine soja) and nine other Glycine species
Source: PLoS One. 2017 Aug 1;12(8):e0182281. doi: 10.1371/journal.pone.0182281 (PMC5538705; doi:10.1371/journal.pone.0182281)

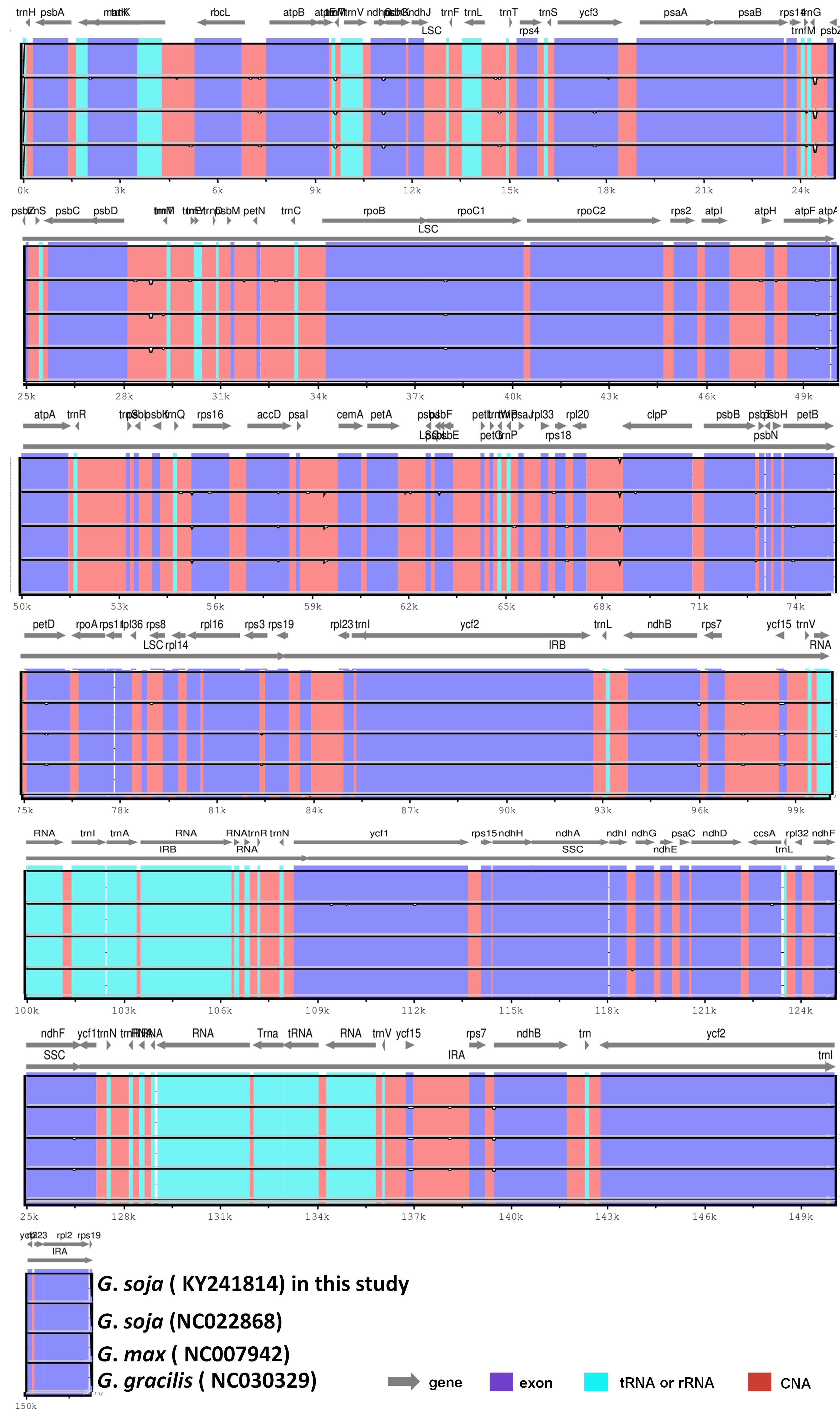

Supplement: S1 Fig — VISTA-based identity plot showing the sequence identity among the ten Glycine species, using G. soja (new) as a reference. Vertical scale indicates the percentage of identity, ranging from 70% to 100%. Horizontal axis indicates the coordinates within the chloroplast genome. Arrows indicate the annotated genes and their transcriptional direction. A thick black line indicates the inverted repeat (IR) regions. (TIF) [file pone.0182281.s006.tif]

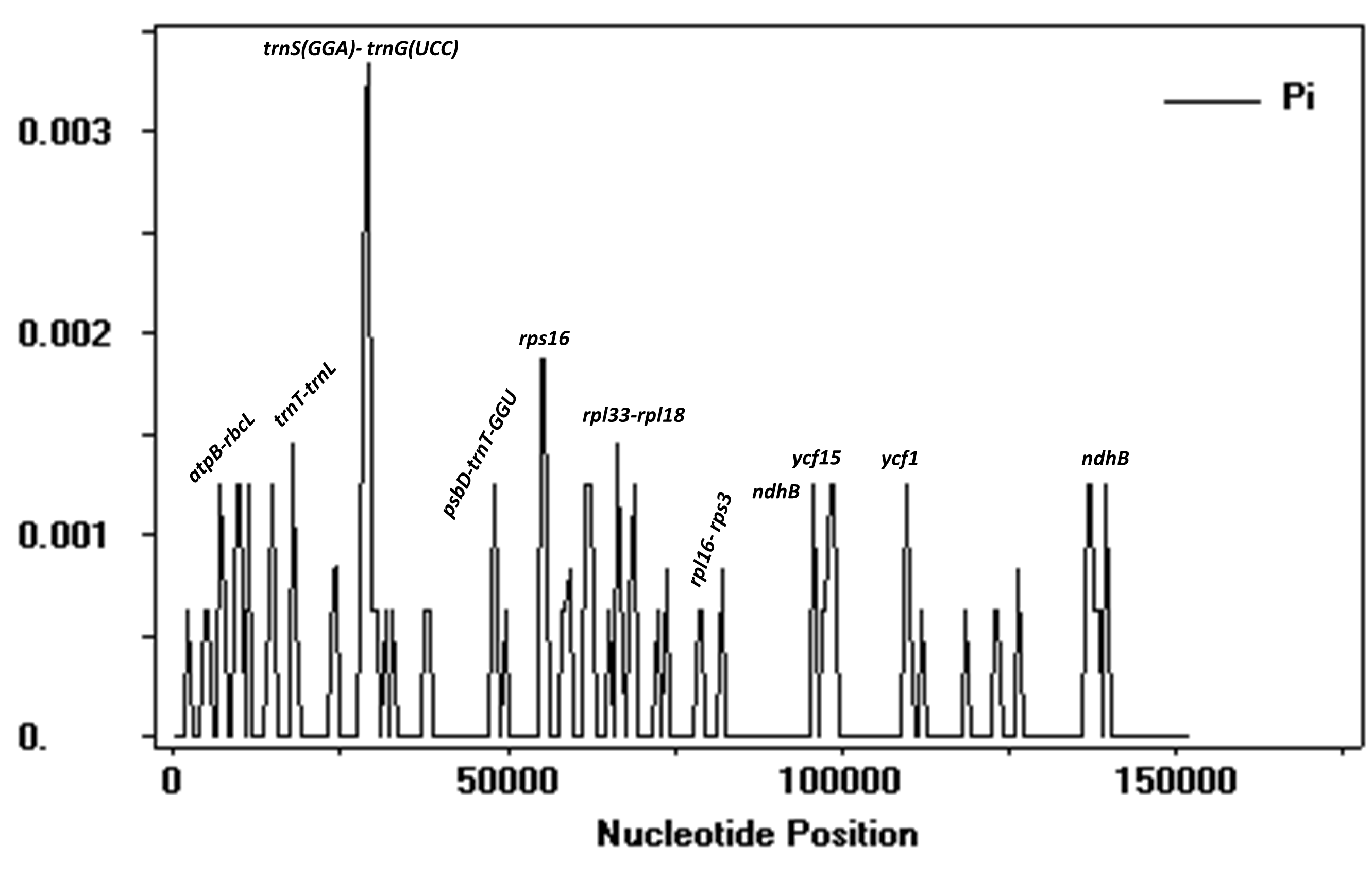

Supplement: S2 Fig — (Window length: 800 bp, step size: 200 bp). X-axis, position of the midpoint of a window; Y- axis, nucleotide diversity of each window. (TIF) [file pone.0182281.s007.tif]

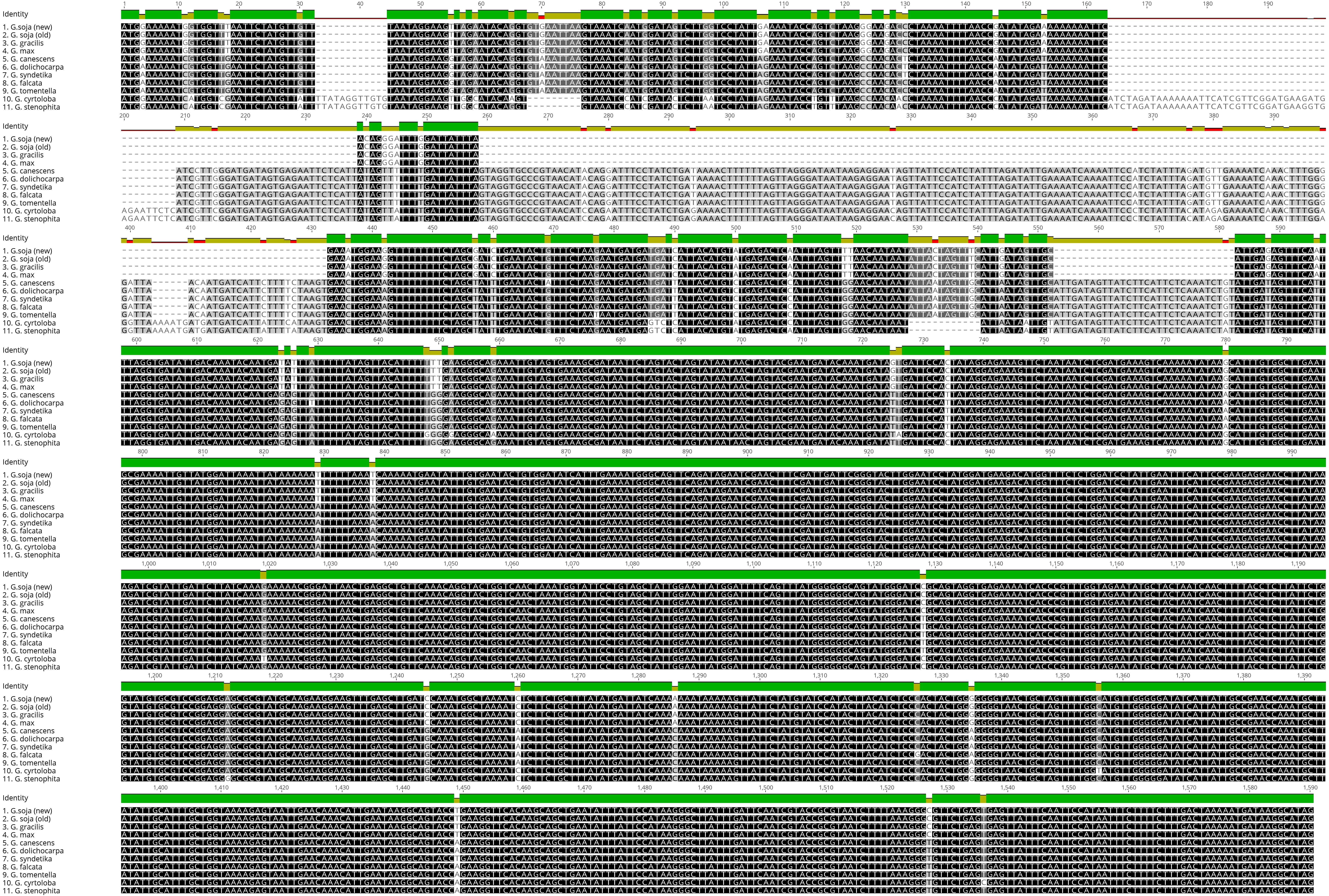

Supplement: S3 Fig — (JPG) [file pone.0182281.s008.jpg]

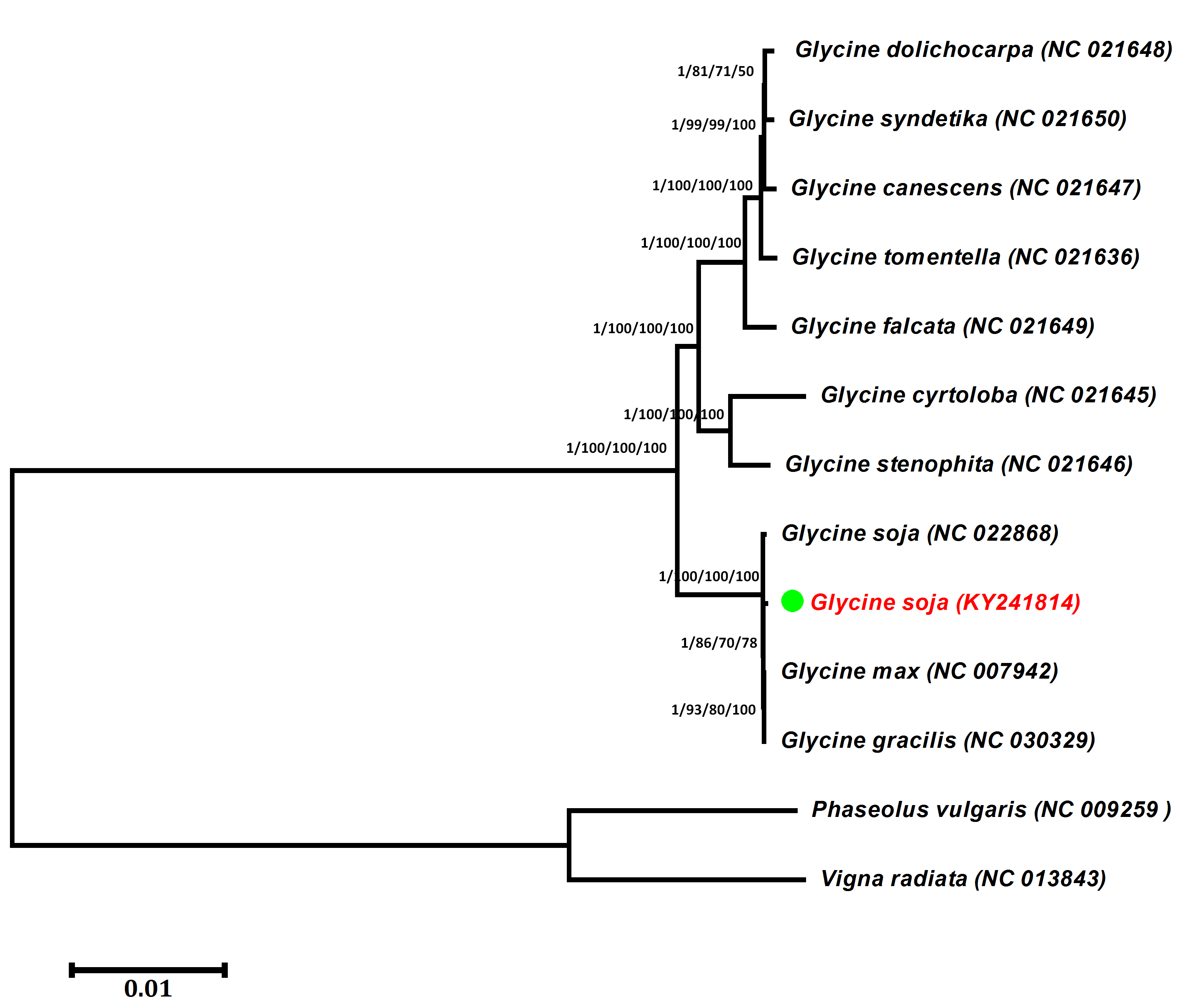

Supplement: S4 Fig — The data from the 76 shared genes were analysed with four different methods: joining-joining (NJ), maximum parsimony (MP), maximum likelihood (ML) and Bayesian inference (BI)). The numbers above the branches are the bootstrap values from the NJ, MP, and ML methods and the posterior probabilities of BI. A red dot represents the position of G. soja (KY241814). (TIF) [file pone.0182281.s009.tif]
